# Helios—Illuminating the way for lymphocyte self‐control

**DOI:** 10.1111/imm.13866

**Published:** 2024-10-01

**Authors:** Iivo Hetemäki, T. Petteri Arstila, Eliisa Kekäläinen

**Affiliations:** ^1^ Translational Immunology Research Program University of Helsinki and Helsinki University Hospital Helsinki Finland

**Keywords:** immunodeficiency diseases, regulatory T cells, T follicular helper cell, transcription factors, T cell

## Abstract

Transcription factor Helios, encoded by the *IKZF2* gene, has an important role in regulatory T cells by stabilizing their suppressive phenotype. While Helios is prominently expressed in regulatory T cells, its expression extends beyond to include effector T cells, follicular regulatory T cells, B cells, and innate‐like lymphocyte populations. Recent characterizations of patients with inborn error of immunity due to damaging *IKZF2* variants coupled with translational research on lymphocytes from healthy individuals, have increased our understanding on Helios' multifaceted role in controlling the human adaptive immune system. A less studied role for Helios beyond the stabilizing of regulatory T cells has emerged in directing effector T cell maturation. In the absence of functional Helios, effector T cells acquire more inflammatory phenotype and are prone to senescence. Loss of Helios expression disrupts the regulation of the germinal centre reaction, often resulting in either hypogammaglobulinemia or B cell autoimmunity. This review summarizes findings from studies in both mice and men offering a comprehensive understanding of the impact of the transcription factor Helios on the adaptive immune system.

## Graphical abstract

Helios, a transcription factor encoded by IKZF2 of the Ikaros transcription factor family, has an important role in regulating adaptive immune response, not only in regulatory T cells but also in effector T cells. Insights from patients with IKZF2 mutations and translational research in healthy individuals underscore how loss of Helios disrupts immune balance, contributing to pro‐inflammatory T cell phenotypes, dysregulated germinal centre reaction, and premature immune senescence. As drugs targeting Helios enter clinical trials, this review synthesizes evidence from both human and murine studies to highlight its impact on sustaining lymphocyte self‐control.

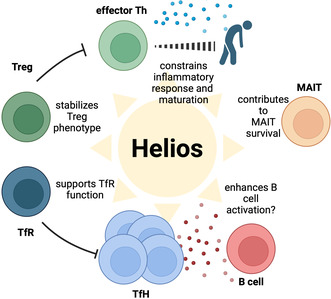

## INTRODUCTION

**FIGURE 1:**
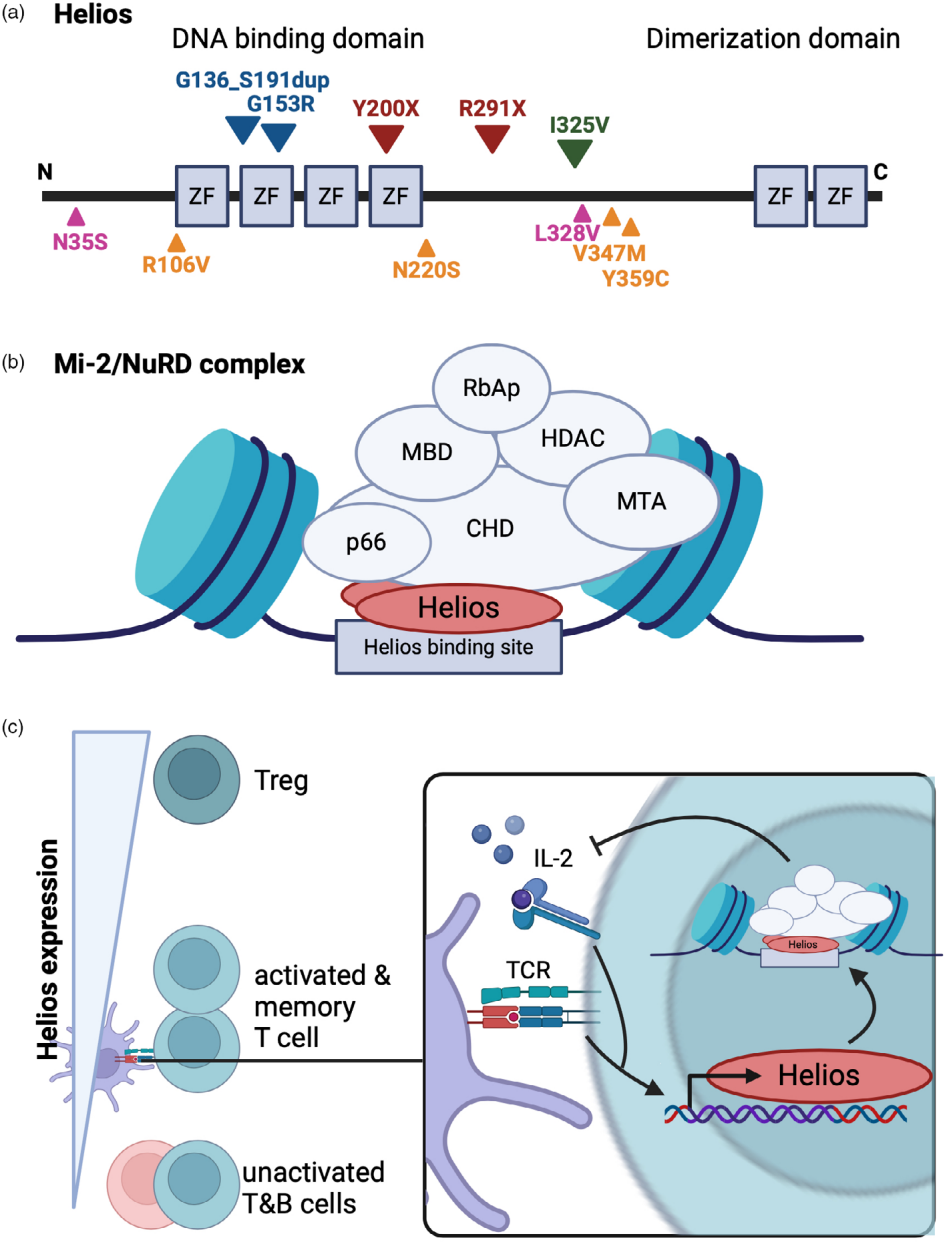
Structure and function of Helios. (a) Schematic presentation of Helios protein structure. Variants that have been associated with immunological perturbations are shown: Heterozygous truncating variants leading to loss of dimerization domain (red), heterozygous missense variants in the DNA‐binding region (blue), homozygous missense variant (green), heterozygous missense variants associated with autoimmune diseases (orange) and detected in patients with anti‐IFN antibodies and autoimmune endocrine disease (pink). (b) Schematic presentation of Mi‐2/NuRD complex. The N‐terminal domain of Helios, binds to specific DNA binding sites, while the C‐terminal domain is responsible for formation of homo‐ and hetero‐ dimers with other Ikaros family members. Helios also binds to proteins involved in the Mi‐2/NuRD complex, including the essential core proteins chromodomain‐helicase‐DNA‐binding protein 3 (CHD3) and metastasis‐associated protein MTA1, that confers both nucleosome remodelling and histone deacetylation. (c) Helios expression in different lymphocytes and its regulation. Helios is upregulated upon T cell receptor (TCR) activation as well as inflammatory cytokine e.g. IL‐2 signalling. Helios directs the Mi‐2/NuRD complex‐mediated nucleosome remodelling that in turn dampens, for example, IL‐2 and IL‐2 receptor‐α chain expression.

The *IKZF2* gene encodes for the transcription factor Helios, named after the sun god of ancient Greek mythology [1]. Helios is a member of Ikaros zinc finger (IKZF) family of transcription factors consisting of five members who all share the same structure of two Krüppel‐like zinc‐finger domains. The N‐terminal domain of Helios, along with other members of Ikaros family, contains structures for DNA binding, while the C‐terminal domain mediates homo‐ and hetero‐dimerization with other Ikaros family members, particularly with Ikaros and Aiolos (Figure 1) [2]. Two less‐studied members of the Ikaros family, Eos, and Pegasus, are expressed in wide variety of tissues, while expression of Ikaros, Helios, and Aiolos is more concentrated in haematopoietic cells, where they have a pleiotropic role in the development and function of the immune system [3].

Helios like other members of Ikaros family function through orchestrating chromatin remodelling through the nucleosome remodelling and histone deacetylase (NuRD) complex, one of the major transcriptional corepressor complexes in mammalian cells (Figure 1b) [4, 5, 6]. The full breadth of genes that Helios directly regulates is unknown, but these include at least repression of IL‐2 and IL‐2 receptor α chain (CD25) expression [7, 8, 9, 10]. Forced overexpression of Helios in human or mouse CD4^+^ T cells and in Jurkat T cell line results in reduced survival of the cells [11, 12].

In peripheral blood, Helios expression is highest in the cells of T cell lineage, but it is detected in B cells [13]. Helios is upregulated after TCR‐mediated activation in T cells, both in αβ and γδ T cells (Figure 1c) [13, 14, 15]. Additionally, inflammatory cytokines control Helios expression with IL‐2, TGF‐β, and TNF‐α enhancing, and IL‐6, IL‐12, and IL‐27 dampening its expression [14, 16, 17, 18]. Public chromatin immunoprecipitation sequencing database identifies especially STAT6, among other transcription factor STATs, as well as RELA and interferon regulatory factor 1 to bind upstream of *IKZF2*. Given the Helios upregulation in response to Th2‐inducing stimuli [19], the IL‐4‐STAT6 signalling might regulate Helios expression, but this topic requires further research. Certain cell subsets, for example, memory T cells, follicular T cells, and particularly regulatory T cells (Treg), constitutively express Helios [13, 14, 19, 20, 21, 22]. Indeed, a lot of research on Helios has concentrated around its effects in Tregs, where it has been proposed to serve as a marker of thymic‐originating tTregs [22].

Somatic mutations in *IKZF2* have long been recognized as contributors to lymphocyte malignancies. In T‐cell leukaemia dominant negative isoforms of *IKZF2* containing intragenic deletion and inversions have been identified [23, 24, 25, 26] and *IKZF2* is also frequently somatically deleted in hypodiploid B‐acute lymphoblastic leukaemias [27]. In contrast, in myeloid leukaemia, Helios is highly expressed in leukaemic stem cells, and its reduced expression in these malignant cells has been suggested to lead to increased differentiation and reduced leukemogenesis [28]. Recently, Helios' role in non‐malignant conditions has been unravelled with several studies describing rare inborn errors of immunity (IEI) due to *IKZF2* germline mutations [13, 29, 30, 31, 32].

Building on the prior work done in knockout mice, recent findings in patients with IEI due to mutations in *IKZF2*, and newest translational research on Helios expression in T cells from healthy individuals across various stages of life, here we synthesize current understanding on Helios and its roles in lymphocytes. We propose that Helios is an important transcription factor regulating function of not just regulatory T cells but also follicular regulatory T cells, and controlling maturation of effector T cells and mucosal‐associated invariant T cells (MAITs). Understanding the broad role Helios has in controlling adaptive immune responses gains particular significance as drugs leading to degradation of Helios are entering clinical trials for treatment of solid tumours [33].

## EFFECTS OF *Ikzf2* IN MICE

In mice, Helios expression is initially detected in haematopoietic cells in earliest haematopoietic sites in the embryo, but also in other tissues including brain, eyes, kidney, and respiratory tract [34, 35, 36]. Subsequently, Helios expression becomes concentrated in T cell lineage and haematopoietic stem cells in adult mice, but it can also be found, for example, in epithelial cells of gut and lung [36]. *Ikzf2* knockout does not appear to disrupt haematopoiesis as the frequency of haematopoietic stem cells, and progenitors of myeloid, B cell, T cell, and erythrocyte lineage are unaltered compared to wild‐type mice [28].

Helios is highly expressed in the murine thymus, especially in the CD4^−^CD8^−^ and CD4^+^CD8^+^ T cells [37] as well as in FOXP3+ thymocytes [22]. Marked Helios upregulation also distinguishes FOXP3‐thymocytes undergoing negative selection [38, 39]. However, effects of *Ikzf2* knockout on thymopoiesis appear limited as frequencies of maturing thymocyte subsets in the thymus of *Ikzf2* ^−/−^ mice did not differ from wild‐type mice [40].

*Ikzf2* ^−/−^ mice do not have a clear immunological phenotype at young age [40]. However, a large fraction of homozygous pups perished for unknown reasons during the first few weeks of postnatal life [40], which has been later speculated to be due to failure to generate peripheral‐induced Tregs [8]. In vivo Helios is upregulated in response to Th2 but not Th1‐inducing stimulus in ovalbumin‐specific transgenic CD4 T cells, but no difference in the overall polarization of T cells was observed in *Ikzf2* ^−/−^ mice or in the polarization of transgenic *Ikzf2*‐deficient T cells in response to antigenic stimuli [19, 20, 40]. When infected with LCMV, both young (2 months) and older (6 months) *Ikzf2* ^−/−^ mice developed autoimmune pathology characterized by immune deposits in kidney and increased fraction of TfH and GC cells, but they were eventually able to clear the infection similar to wild type mice [41]. Only older *Ikzf2* ^−/−^ mice at the age of 6–8 months spontaneously developed an autoimmune phenotype resembling some features of systemic lupus erythematosus (SLE) with infiltration of lymphocytes to non‐lymphoid organs, production of autoantibodies, and glomerulonephritis [41, 42].

## INBORN ERRORS IN IMMUNITY DUE TO *IKZF2* VARIANTS

It was not until recently that patients with germline mutations in *IKZF2* were described. These patients display varying clinical phenotypes (Table 1) that are mainly distinct from *Ikzf2* ^−/−^ mice, but some common patterns of immune dysregulation emerge. Notable for *IKZF1* (encoding Ikaros) mutations is that the clinical phenotype of patients is highly variable depending on the mutation site and subsequent depth of loss of Ikaros function [43]. Similar pattern is emerging also for *IKZF2*.

**TABLE 1:** Characteristics of patients with *IKZF2* mutations.

	Dimerization		DNA binding		Other				
Mutation	p.Y200X	p.R291X	p.G153R	p.G136_S191dup	p.I325V	p.V347M	p.R106W	p.Y359C	p.N220S
Infections	+	−	+	+	+	+	+		
Failure to thrive	−	−	+	+	+				
Autoimmune cytopenias	−	+	−	+	−	+		+	+
Developmental abnormalities	−	−	+	+	−				
Hypogammaglobulinemia	+/−		+	−	+	+	+		
Lymphoma	−/+								
HLH						+	+		

Abbreviation: HLH, hemophagocytic lymphohistiocytosis.

We have described two patients from a single pedigree, with a heterogenous *IKZF2* p.Y200X truncating variant leading to loss of Helios' dimerization domain (Figure 1a, red) [13]. The p.Y200X variant was translated to RNA and produced in a cell line. However, the interactome analysis indicated decreased interactions with several proteins including those involved in sumoylation, known to affect the stability of Ikaros family members, and no variant protein was detectable from patient cells. Similar findings have been reported in patients with dimerization defective mutation in *IKZF1* [44], and we suggest that the p.Y200X variant caused dimerization defective loss of function of *IKZF2*.

Both patients with *IKZF2* p.Y200X had recurrent upper respiratory infections and mucosal *Candida albicans* infections, lymphadenopathy, and one had a history of Hodgkin's lymphoma and the other hypogammaglobulinemia. Their T cells are skewed towards mature phenotype with accumulation of terminally differentiated CD27^−^CD28^−^CD57^+^ T cells and, in the older patient, there was a failure of T cell proliferation upon stimulation. Ex vivo both the transcriptome analysis and chemokine receptor expression indicated Th1 polarization, and after in vitro TCR stimulation the patient T cells had increased IL‐2 and IFN‐gamma production in both effector and regulatory T cells and the transcriptome signature indicative of enhanced IL‐2 and IFN‐gamma signalling. The frequency of circulating Tregs was slightly reduced in patients, but in suppression assay, the Tregs suppressed effector T cell proliferation comparably to healthy controls. The frequency of circulating CXCR5^+^ follicular T helper cells was markedly reduced, while analysis of patient lymph node samples revealed follicular hyperplasia with an accumulation of proliferating follicular T cells. In line with disrupted T cell control in lymph nodes and germinal centres, we could detect several B cell aberrations. One of the patients had a high frequency of activated B cells, but a decreased amount of switched memory B cells and plasmablasts in line with hypogammaglobulinemia while in the other had developed high titers of neutralizing antibodies against cytokines including type I interferons. Finally, the patients had low frequency of MAITs both in peripheral blood and in gut biopsies.

Shahin et al. described [30] a patient with *IKZF2* p.R291X variant causing also a truncated protein with similar loss of dimerization domain (Figure 1a, red). The patient, a 16‐year‐old female, suffered from early‐onset SLE. Single‐cell transcriptomic analysis indicated activation of both monocytes and T cells in the patient with upregulation of genes associated with T cell exhaustion. The patient suffered from hypogammaglobulinemia with reduced number of circulating TfH but exhibited otherwise very little T cell abnormalities.

Whereas the *IKZF2* p.Y200X and p.R291X appear to be defective dimerization mutations with ‘milder’ loss of Helios function, recently two children with heterozygous mutations in the DNA binding region of *IKZF2* (p.G136_S191dup and p.G153R; Figure 1a, blue) were described with both more severe clinical and immunological phenotype [32]. These patients failed to thrive and suffered from infections already from birth. They had T cell lymphopenia with low T cell receptor (TCR) excision circle counts indicative of failed generation of T cells. The first patient developed autoimmune hemolytic anaemia at the age of 2 months, while the other had low B cells, low total IgG, and poor responses against tetanus and pneumococcal polysaccharide vaccines. Both patients also experienced developmental issues, such as neurocognitive impairment and craniofacial abnormalities impacting eye development and bilateral sensorineural hearing loss that align with observations in *Ikzf2* ^−/−^ mice and sites of *IKZF2* expression during embryogenesis [34, 35, 36, 40].

A patient with homozygous missense mutation *IKZF2* p.I325V (Figure 1a, green) [29] affecting area between the two zing‐finger domains of the protein had intact DNA binding and dimerization of Helios but interactome analysis indicated that the variant had reduced interactions with protein partners of the Mi‐2/Nurd complex. The patient was an adolescent from a consanguineous family who had presented with a failure to thrive, hypothyroidism, history of recurrent infections, and hypogammaglobulinemia. The immunophenotype resembled the cases with truncating variants with higher relative abundance of effector CD8^+^ cells with increased CD57 expression and reduced proliferation of T cells upon stimulation. Transcriptome analysis revealed increased inflammatory phenotype of both monocytes and T cells. The frequency of Tregs was reduced with an increased IL‐2 and IFN‐γ production, whereas in effector T cells IL‐2 production was reduced and production of IFN‐γ unaltered compared to healthy controls. The patient had failure of B cell maturation with increased fraction of transitional B cells and CD38lowCD21low B cells and decreased transcriptional signature associated with BCR signalling. The patient had also decreased frequency of MAIT cells.

Additionally, heterozygous missense *IKZF2* variants (Figure 1a, orange) have been found in five patients with clinical phenotypes consisting of SLE, immune thrombocytopenia, or EBV‐associated hemophagocytic lymphohistiocytosis [30]. Two patients with missense *IKZF2* variants (Figure 1a, pink) were identified after screening of patients with autoimmune endocrine disorders with anti‐IFN antibodies [31].

In summary, there is large phenotypic variation among published patients with *IKZF2* variants that most likely stems from the extent of Helios functional deficiency resulting from the different mutations. The limited number of identified patients makes it challenging to draw definite conclusions about the processes influenced by Helios. However, few common features emerge, consistent with findings from mouse models: premature T cell senescence, reduction in frequency of Tregs, and dysregulated B cell response (Table 2). In the following chapters, we will focus on known functions of Helios in these cells.

**TABLE 2:** Immunological characteristics of patients with *IKZF2* mutations.

		Human				Mouse
		Dimerization		DNA binding		
	Mutation	p.Y200X	p.R291X	p.G136_S191dup p.G153R	p.I325V	Various
Effector T cells		‐	‐	↓	‐	
	Senescence	↑	↑		↑	
	IL‐2 secretion	↑			↓	↓
	IFNy secretion	↑			‐	↑
Treg						
	% in circulation	↓	‐		↓	↓
	suppression	‐				‐/↓
	IL‐2 secretion	↑	↑		↑	↑
TfH						
	% in circulation	↓↓	↓		‐	
	% in lymph node	↑↑				↑↑
B cells		‐	‐	↓	↓	
	Transitional	↑			↑	
	Class‐switched	‐/↓	↓		↓	
	IgG levels	‐/↓	‐	‐/↓	↓	‐
NK cells		‐	↓		↓	
Type I IFN signalling		↑/‐			↑	

*Note*: ↑ = elevated, ↓ = decreased, − = normal.

## HELIOS STABILIZES THE NON‐INFLAMMATORY PHENOTYPE OF REGULATORY T CELLS

Regulatory T cells stand out as the T cell population with the most notable constitutional expression of Helios [11], but the significance of Helios expression in Tregs is debated. Helios has been suggested to be a marker for thymus‐derived natural Tregs (tTreg) [22]. This is supported by the observations in mice, that (1) the Foxp3^+^ T cells that are generated in the thymus during the first week of postnatal life uniformly express Helios, (2) Helios^−^ Tregs only reach their maximum frequency after weaning, and (3) Helios expression was not detected in peripherally induced Tregs (pTreg)s in the early models studying their induction (reviewed by Thornton and Shevach [45]).

Other studies contest this theory. Later mouse models of Treg induction have identified Helios expression in pTregs [16, 46, 47]. Furthermore, in human fetal naive non‐Treg cells, a moderate basal Helios expression, along with other features of Tregs transcriptome, was observed [8]. These cells had increased chromatin accessibility to the *IKZF2* gene and a fraction of other genes described to be part of Treg‐spesific epigenome. In vitro induction of Tregs from fetal naive T cells led to upregulation of Helios beyond the baseline and the high Helios expression was sustained, which differed from the case in adult‐derived induced Tregs. Additionally, a prior study in mice suggested that upregulation of Helios would be a characteristic for peripheral effector T cells driven towards tolerance [39]. The significant percentage of Helios expressing Tregs detected in newborn mice might not solely result from Helios expression limited to tTregs, but rather reflects the overall predisposition of the fetal immune system towards tolerance, leading to high Helios expression in both tTregs and pTregs.

Efforts to uncover the ancestry of Tregs with or without Helios expression have also employed TCR repertoire analysis. In mice with a restricted TCR repertoire, the TCR repertoires of Helios^+^Tregs and Helios^−^Tregs were similar [47]. In Helios reporter mice, however, overlap between the TCR sequences of Helios^+^Tregs and Helios^−^Tregs isolated from the same lymph node was 13% compared to 9% between Helios^−^Tregs and naive T cells, and 27% between Helios^+^Tregs and naive T cells [48]. In samples from the lamina propria and mesenteric lymph nodes from patients with ulcerative colitis, Helios^−^Tregs had more overlap with Helios^+^Tregs than with FOXP3^−^ effector T cells [49]. Analyses of human secondary lymphatic tissue Tregs associated high Helios expression with distinct TCR repertoire that predominantly differed from that of effector T cells suggesting that these cells were tTregs [50, 51]. On the other hand, low Helios expression in Tregs was linked to TCR repertoire shared predominantly with TfH cells although these Helios^−^Tregs also shared some clones with Helios^+^Tregs [50, 51]. These somewhat mixed results link Helios^−^Tregs ancestry to both Helios^+^Tregs as well as effector T cells.

Helios expression is associated with functional differences in Tregs. Human secondary lymphatic tissue Helios^+^ and Helios^−^ Tregs exhibit distinct transcriptional expression profiles, with Helios^−^ Tregs displaying a transcriptome more akin to effector T cells [50, 51]. Helios expression stabilizes the non‐inflammatory phenotype of Tregs and prevents IL‐2 production by epigenetic silencing [7, 8, 9]. In selective knockout models in both mice and human cell cultures, as well as in human memory Tregs ex vivo, Helios^−^Tregs display heightened proinflammatory cytokines production and lower FOXP3 expression compared to their Helios‐expressing counterparts [9, 52, 53]. These characteristics are typical for unstable Tregs, and the loss of Helios may cause Treg conversion to effector T cells [52]. Conversely, knockdown of Helios expression in human fetal naive T cells impeded their in vitro induction to Tregs [8].

Both the patient with homozygous p.I325V and the patients with heterozygous p.Y200X mutation had a reduced number of Tregs with increased Treg IL‐2 production that might indicate Treg instability [13, 29]. Analysis of TSDR demethylation within the FoxP3 locus associated with Treg stability, revealed comparable results in Tregs of a patient with p.Y200X mutation and healthy controls [13] in line with results from comparison of Tregs of *Ikzf2* ^−/−^ and wild‐type mice, and in sorted human Helios^−^Tregs compared to Helios^+^Tregs [8, 42]. However, a study using mice with Helios‐GFP reporter depicted that Helios^−^Tregs had slightly lower demethylation than Helios^+^Tregs [48]. These observations suggest that high Helios expression marks the Tregs with stable suppressive phenotype both in mice and humans, while Helios‐low Tregs are more inclined to acquire effector functions, likely due to epigenetic differences caused by Helios.

The differences in Tregs with or without Helios extend to their ability to suppress inflammation. Indeed, *Ikzf2* knockdown with the Foxp3 promoter in mice is sufficient to induce autoimmunity [41, 42]. In vitro, surprisingly, suppressive capability of Tregs in *Ikzf2* knockout mice was intact [40, 42]. Also in human individuals with defective Helios expression, in vitro Treg suppression of effector T cell proliferation after TCR stimulation was comparable to those of healthy control [13]. In vivo, transfer of Helios^−^Tregs, either isolated from Helios reporter mice or from *Ikzf2* knockdown mice, were equal to Helios^+^Tregs in their ability to suppress development of inflammatory bowel disease, but they were inferior in inhibiting the autoimmune disease that follows from transfer of effector T cells from Scurfy mice lacking functional Foxp3 into *Rag* ^−^^/^^−^ recipients [41, 42, 48]. While in vitro suppressive capacity of Helios^−^Tregs appears unaffected, intact Helios expression is required for full suppressive capacity of Tregs in vivo.

Taken together, studies in both mice and men indicate that, while Helios expression might not be exclusively limited to tTregs, Helios expression in Tregs appears to associate with thymus‐derived Treg origin. Tregs without Helios have slightly lower suppressive capacity and they can derive from both tTregs as well as iTregs. In Tregs, Helios stabilizes the non‐inflammatory phenotype and prevents IL‐2 production.

## LACK OF HELIOS LEADS TO FOLLICULAR T HELPER CELL EXPANSION

Studies done in both mice and humans highlight the essential role of Helios in regulation of the germinal centre reaction. Helios is expressed by both TfH and TfR. In mice, Helios is upregulated in TfH cells in vivo in response to stimulation, but *Ikzf2* knockout does not affect effector T cells ability to differentiate to TfH cells, nor the ability of transgenic Helios^−^ TfH cells to provide help for B cells after adoptive transfer to *SAP* ^−/−^ recipient, where the endogenous T cells are unable to provide help to B cells [19, 20]. In humans Helios expression in lymph node TfH cells ex vivo is higher than the one observed in non‐follicular T effector cells, but not as high as in TfRs, which express Helios at levels similar to Tregs [13, 21]. Recently number of studies have dissected the heterogeneity of human TfR population and identified two subgroups: ‘natural’ TfRs with high Helios expression that develop from Tregs and have superior suppressive capacity in vitro, and ‘induced’ TfR with low Helios but high Aiolos expression that seem to predominantly originate from TfH cells [50, 51].

In both mice and humans, Helios defect results in excessive accumulation of TfH in lymph nodes. Both germline knockout of *Ikzf2* or selective knockout with Foxp3 promoter in mice resulted in increased germinal centre formation and accumulation of Tfh and GC B cells in lymph nodes [41, 42]. TfH cell frequency expanded to an enormous 75‐fold in the spleen in mice with *Ikzf2* knockout with FoxP3 promoter compared to wild‐type mice [42]. Also, the number of TfR cells was increased compared to wild‐type mice, but not in the magnitude of TfH. Notably, TfH accumulation in the lymph node was also observed after immunization in mice with heterozygous knockout of *Ikzf2* with Foxp3 promoter [42]. In a reminiscent pattern, the analysis of lymph nodes of patients with *IKZF2* p.Y200X mutation indicated accumulation of cells with TfH phenotype and Ki‐67 expression was increased indicating rapid proliferation. In contrast, the circulating follicular T helper cells were almost completely absent in the patients with p.Y200X as well as in the patient with p.R291X perhaps reflecting retention of TfH cells in the secondary lymphatic tissues [13, 30].

Changes in the T cell compartment of the germinal centre, reminiscent of those seen in Helios deficiency, are also seen secondary to other mutations affecting function of TfRs: in mice knockdown of *Foxp3* or *Ctla‐4* lead to accumulation of both TfH and TfR in the lymph nodes [54, 55]. Patients with a CTLA‐4 deficiency or IPEX have chronic lymphadenopathy and their B cell phenotype is similar, albeit often more severe, to those observed in many patients with Helios mutations: increased fraction of transitional B cells and CD21^lo^CD38^lo^ chronically activated B cells, and often failure of class‐switching and progressive loss of B cells culminating in hypogammaglobulinemia [56, 57, 58]. Notably in all these IEI affecting Treg function, dysregulated germinal centre reactions have also been described to lead to production of neutralizing anti‐IFN antibodies [13, 31, 59].

In summary, the TfR intrinsic role of Helios appears to be crucial for effective regulation of the germinal centre reaction, as *Ikzf2* knockout with FoxP3 promoter is sufficient to cause dysregulation of the germinal centre reaction. Helios deficiency can lead to failure of B cell maturation and hypogammaglobulinemia [13, 29, 30] or B cell autoimmunity. SLE, a prototypical disease of autoantibody production, or autoimmunity reminiscent to SLE is seen in both fractions of patients with *IKZF2* variants and in older *Ikzf2* ^−/−^ mice [30, 41, 42], and polymorphism in Helios have been identified as a risk factor for SLE in humans [60, 61].

## HELIOS AND B CELL INTRINSIC EFFECTS?

Is the B cell pathology in both *Ikzf2* knockdown mice and patients with the *IKZF2* variants solely mediated by changes in T cells? Though Helios expression in B cells is lower than in T cells, it is still present, and the expression increases in hand with B cell maturation in humans [13]. A study examining the role of EBV infections in the pathogenesis of SLE in humans found that the *IKZF2* was one of the most differentially expressed genes in EBV‐infected B cells compared to normal B cells [62], but little more is known about regulation of Helios expression in B cells or its effects on human B cells. In mice, ectopic expression of Helios with Ig promoter resulted in prolonged survival and enhanced proliferation of B cells. leading to hyperresponsiveness to antigenic stimulation [63]. Conversely, in chicken immature B cell line DT40 *Ikzf2* knockout led to resistance to PMA stimulation [64], and in the patient with the p.I325V mutation, the transcriptional profile of the B cells suggested reduced BCR activation [29]. Helios might have direct cell‐intrinsic effects on B cell maturation that warrant more thorough studies in the future.

## LOSS OF HELIOS IS ASSOCIATED WITH A LOSS OF MUCOSAL‐ASSOCIATED INVARIANT T CELLS

Constitutive Helios expression has also been described in human innate‐like lymphoid cells including unconventional CD8^+^ T cell population [65], natural killer (NK) cells [66], and mucosal‐associated invariant T cells [67]. Recently an unconventional CD8^+^ T cell population characterized by KIR and Helios expression was described in human thymus, blood, and intestines [65]. These cells have been suggested to be suppressive CD8‐positive regulatory cells and their frequency was elevated in several autoimmune diseases but also in viral infections [68]. The frequency of CD15^+^ CD56^dim^ NK cells was reduced in patients with the *IKZF2* p.I325V and p.R291X mutations [29, 30], but comparable to healthy controls in patients with the *IKZF2* p.Y200X [13]. NK cell cytotoxicity was tested in one patient with the *IKZF2* p.V327M variant with chromium release test, yielding normal results [30].

MAIT cells play a role in bacterial defence on mucosal surfaces and also recognize fungal metabolites in vitro [67]. MAITs undergo development in the thymus as a minor population, and upon egress from the thymus, they rapidly expand and acquire effector memory—like phenotype [69]. We could detect Helios expression from over 90% developing MAIT cells isolated from human thymuses [13]. The human peripheral blood MAITs were already predominantly Helios positive, but stimulation with *Escherichia coli*, *C. albicans*, or PMA led to an increase in Helios expression [13]. Patients with the p.Y200X mutation had profound reduction in frequency of MAITs in peripheral blood as did the patient with p.I325V mutation [13, 29]. While the expression of activation and tissue retention marker CD69 was higher in MAITs of patients with the p.Y200X there was no signs of accumulation of MAITs in gut biopsy samples and the patients had recurrent mucosal *Candida albicans* infections [13]. Reduction in circulating MAITs is not uncommon finding in IEI with immune dysregulation and the decrease in MAITs in Helios deficient patients might be attributed to increased activation by pro‐inflammatory cytokines and subsequent consumption [70, 71, 72]. However, considering constitutional Helios expression in MAITs throughout their development and the profound decrease in both blood and gut detected in the patients, Helios might also have a non‐redundant role in maintenance of MAITs.

## HELIOS ATTENUATES INFLAMMATORY PHENOTYPE OF EFFECTOR T CELLS IN CELL‐INTRINSIC MANNER

Helios suppresses pro‐inflammatory responses not just in Tregs but also directly in effector T cells: high Helios expression is associated with dampened effector T cell response, while Helios silencing enhances the pro‐inflammatory response of effector T cells. In humans, high Helios expression in fetal naive T cells was associated with reduced IFN‐γ secretion [8], and adult CD8^+^ T cells with high Helios expression had both reduced IFN‐γ secretion and T cell degranulation capability compared to their counterparts with low Helios expression [73]. Also inhibiting the normal upregulation of Helios in effector T cells upon TCR stimulation in vitro, either in adult effector CD4^+^ T cells with siRNA or selective molecular glue degrader of Helios or in fetal naive T cells with CRISPR‐Cas9 silencing, resulted in heightened IFN‐γ secretion [8, 13, 33, 74].

Notably, in clinical samples from lymphoma patients high frequency of Helios expressing CAR‐T cells was a marker of poor prognosis. Conversely, Helios silencing improved CAR‐T cell killing potency against glioblastoma cells both in vitro and in mouse xenograft models [74]. This improvement was accompanied with pro‐inflammatory and effector‐associated genetic signatures in CAR T cells, including IFN‐γ upregulation [74]. Also, administration of Helios degrader enhanced the anti‐tumour immune response of humanized mice towards xenograft breast cancer [33]. Collectively, these studies indicate that Helios has a cell‐intrinsic role in effector T cells in suppressing pro‐inflammatory response, especially IFN‐γ secretion and Th1 response. Indeed, in the patients with p.Y200X mutation, T helper cells exhibited a proinflammatory phenotype with clear signs of Th1 polarization both ex vivo and in vitro [13].

To this backdrop, it is an interesting finding that ageing is associated with decrease in Helios expression of human naive CD4^+^ T cells [10]. This reduction appears to account for increased CD25 expression in the naive T cells, along with altered activation‐induced chromatin changes that favour pro‐inflammatory effector cell differentiation [10]. Abrogation of Helios from human naive T cells that were subsequently activated and transferred to chimeric mice carrying human synovial tissue resulted in enhanced T cell activation, effector differentiation and tissue‐invasiveness in vivo [10]. Decline in Helios expression with ageing might be in part responsible for increased disposition of the elderly to autoimmune diseases.

## HELIOS DEFICIENCY LEADS TO ACCUMULATION OF TERMINALLY DIFFERENTIATED EFFECTOR T CELLS

Illustrating the dynamic role Helios has in modulating pro‐inflammatory T cell response, its expression increases on average with T cell maturation and is especially high in terminally differentiated effector T cells and those cells that have lost their full response potential [13, 14]. In the LCMV murine infection model of T cell exhaustion, Helios emerges as one of the most important transcription factors differentiating exhausted virus specific T cells from naive and memory cells, particularly among CD4^+^ cells [75, 76]. Similarly, in mice with transgenic TCR specific for a gastric autoantigen, functionally anergic peripheral T cells in gastric lymph nodes expressed high levels of Helios [39]. In humans, high Helios expression correlates with characteristics of T cell exhaustion in CD4^+^ memory cells in HIV patients [77] as well as in human tumour infiltrating CD8^+^ T cells [52, 78]. While in naive CD4^+^ T cells, ageing was associated with decline of Helios expression, the CD8^+^ T cells accumulate with ageing a population marked by co‐expression of Helios and co‐inhibitory receptor TIGIT that had typical features of T cell senescence: reduced proliferation, downregulation of co‐stimulatory receptors CD27 and CD28 and expression of CD57 [79]. Helios expression appears thus to be characteristic for both CD4^+^ and CD8^+^ T cells that have acquired features of reduced responsiveness in T cell exhaustion or senescence.

Whereas silencing of Helios seems to increase inflammatory potential of effector cells in the short term, in the long run, Helios deficiency seems to usher premature accumulation of senescent T cell subsets that normally have high Helios expression, and result in the reduction of the overall responsiveness of T cells. The T cell phenotype of Helios deficient patients was excessively aged for their years: both patients with heterozygous p.Y200X and with homogenous p.I325V mutation had increased fraction of CD27^‐^CD28^‐^ with increased CD57 expression, and though the production of proinflammatory cytokines by T cells seemed intact, their capability to proliferate was reduced [13, 29] manifesting hallmarks of senescent T cells. Also in mice, Helios^−^ CD4^+^Foxp3^−^ ovalbumin specific effector T cells produced a normal primary response after adoptive transfer, but these cells failed to expand upon secondary antigen exposure in contrast to their Helios sufficient counterparts [20].

**FIGURE 2:**
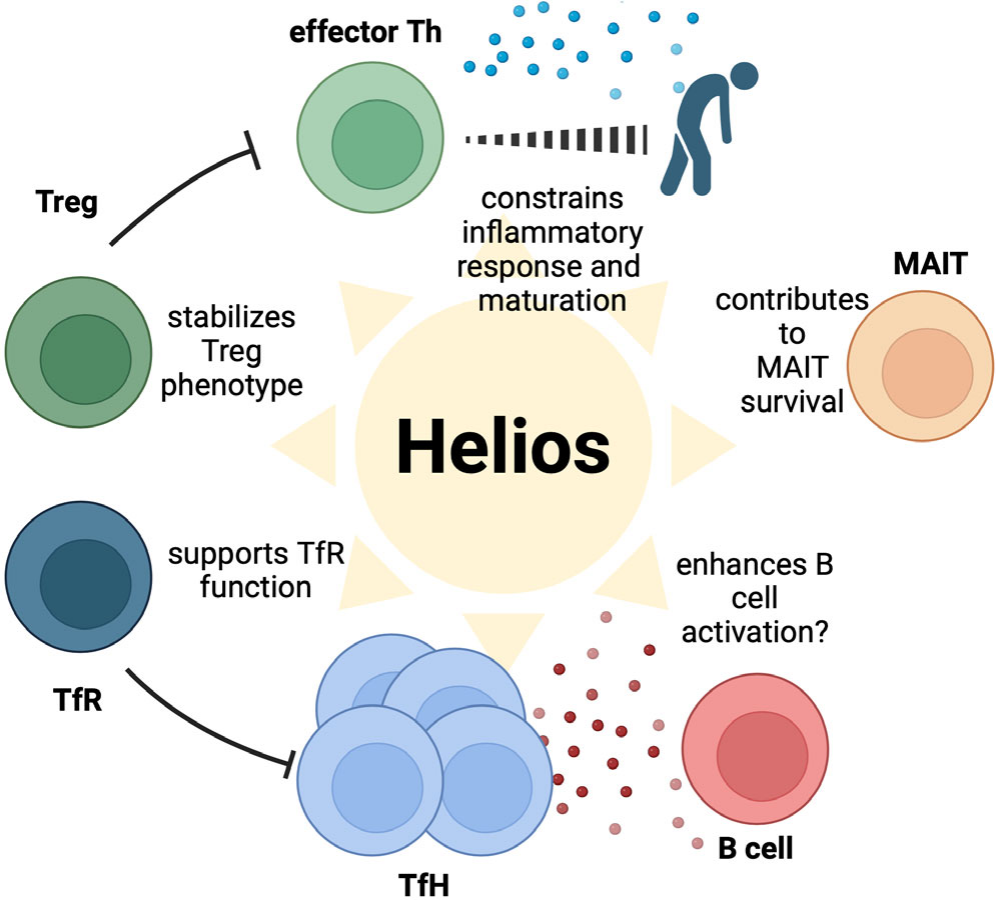
Role of Helios in regulating lymphocyte self‐control.

In conclusion, Helios appears to function as a ‘break’ restraining the responsiveness of effector T cells. A parallel to Helios' dual role in both effector and regulatory T cells can be drawn between CTLA‐4 and FOXP3. While all three have constitutional high expression in Tregs, they are also upregulated in effector T cells upon activation, resulting in attenuation of inflammatory responses [57, 80, 81, 82]. If the Helios ‘break’ is missing, as in the patients with IEI with *IKZF2* mutations, the T cells live a taxing life akin to one of a young rock‘n'roll star—intense proinflammatory T cell responses, but premature ageing of T cells to fully matured, even senescent phenotype (Figure 2).

## CONCLUSIONS

The immune system faces a constant challenge in generating controlled and appropriate immune responses, striking a delicate balance between adequacy and avoiding excess. The transcription factor Helios, expressed in a wide variety of lymphocyte populations, plays a pivotal role in this essential self‐control effort of the adaptive immune system (Figure 2). Particularly in T cells, the physiological increase in Helios expression upon both activation and T cell maturation illustrates its role in fine‐tuning immune response. The decline in Helios expression of the naive effector CD4^+^ T cell observed in the old can, at least partially, explain why this delicate control is lost, contributing to chronic low‐grade inflammation associated with human ageing. Better understanding of pathways leading to upregulation and upkeep of Helios expression might lead to future therapeutic targets aimed at mitigating inflammaging.

The limited number of identified human patients with damaging *IKZF2* variants suggests that the more severe mutations affecting the gene's function might be lethal as observed in a large fraction of newborn *Ikzf2* ^−/−^ mice. The combined immunodeficiency evident at birth in the few identified patients with mutation in the DNA binding region underscores the detrimental impact of severe Helios deficiency. However, the clinical phenotype of patients with *IKZF2* variants varies, mirroring the mutation site and subsequent variable impact on Helios protein's functions, and in some patients, clinical signs of immune dysregulation are subtle and manifest at older age. Treatments in advanced solid tumours targeting Helios degradation may not be without side effects, but these will likely develop gradually if some of the protein's function is reserved. Lastly, knockdown of Helios seems to be a potential target to enhance the effector capability of, for example, tumour targeting T cells in the short run but this must be balanced with potential long‐term consequence of heightened T cell senescence.

## FUNDING INFORMATION

This work was supported by the Finnish Medical Foundation, the Päivikki and Sakari Sohlberg foundation, Sigrid Jusélius Foundation, and EJPRD JTC2019 & Academy of Finland (308912 and 308913).

## CONFLICT OF INTEREST STATEMENT

The authors declare no conflict of interest.

## Data Availability

Data sharing is not applicable to this article as no new data were created or analyzed in this study.
